# Development and validation of a diagnostic nomogram for Wagner grade≥2 diabetic foot ulcers in hospitalized patients with type 2 diabetes

**DOI:** 10.3389/fendo.2026.1792343

**Published:** 2026-04-21

**Authors:** Jinying Zhang, Xuesong Jiang, Yan Li, Jiayu Lin

**Affiliations:** 1 Department of Geriatrics, The Second Affiliated Hospital of Fujian Medical University, Quanzhou, China; 2 Department of Orthopedics, Quanzhou Southeast Hospital, Quanzhou, China; 3 Department of Endocrinology, The Second Affiliated Hospital of Fujian Medical University, Quanzhou, Fujian, China

**Keywords:** diabetic foot ulcer, logistic regression, nomogram, thromboelastography, type 2 diabetes

## Abstract

**Background:**

Diabetic foot ulcers (DFUs) represent a severe and prevalent complication of diabetes, contributing to substantial disability and elevated mortality. This study aimed to develop and validate a diagnostic nomogram for Wagner Grade ≥2 DFUs in hospitalized patients with type 2 diabetes (T2DM).

**Methods:**

This retrospective cohort study included 510 hospitalized patients with T2DM treated at the Second Affiliated Hospital of Fujian Medical University between January 2023 and December 2025, of whom 248 had Wagner Grade ≥2 DFUs. Patients were randomly divided into a training set (n=357) and an internal validation set (n=153) in a 7:3 ratio. Univariate and multivariate logistic regression analyses were performed to identify independent risk factors and construct a diagnostic nomogram. Model performance was evaluated using receiver operating characteristic (ROC) curves, calibration curves, decision curve analysis (DCA), and clinical impact curves. External validation was performed in an independent cohort of 154 patients from Quanzhou Southeast Hospital, including 86 with Wagner Grade ≥2 DFUs.

**Results:**

The nomogram incorporated four independent predictors: Angle α, K time, platelet count (PLT), and lymphocyte count. The model exhibited excellent discrimination in the training set (area under the curve [AUC] = 0.940, 95% confidence interval [CI]: 0.916–0.965) and internal validation set (AUC = 0.914, 95% CI: 0.870–0.956), with modest discrimination in the external validation set (AUC = 0.690, 95% CI: 0.604–0.775). Calibration curves demonstrated strong concordance between predicted and observed probabilities. DCA and clinical impact curves confirmed substantial clinical utility across all cohorts.

**Conclusion:**

This nomogram, integrating thromboelastography parameters and hematological indicators, provides a practical tool for identifying the presence of Wagner Grade ≥2 DFUs in hospitalized patients with T2DM, supporting early risk stratification and informed clinical decision-making.

## Introduction

Diabetes mellitus is a chronic metabolic disorder characterized by hyperglycemia, affecting approximately 537 million adults worldwide in 2021, with projections rising to 783 million by 2045 (1). Primarily T2DM, it is associated with microvascular and macrovascular complications, including retinopathy, nephropathy, neuropathy, and peripheral arterial disease. Diabetic foot ulcers (DFUs) are among the most burdensome, leading to substantial morbidity, mortality, and healthcare costs (2).

Globally, DFUs affect 6.3% of individuals with diabetes, with higher prevalence in males (4.5%) than females (3.5%), and slightly elevated in type 2 (6.4%) versus type 1 diabetes (5.5%). Lifetime risk reaches up to 34%, impacting 18.6 million people annually and imposing significant economic burdens through prolonged hospitalizations and treatments (3–5). Prevalence is notably higher in low-resource settings, exceeding 18% in some T2DM populations, due to poor glycemic control, limited healthcare access, and inadequate patient education.

DFUs are classified using systems like the Wagner grading, which assesses lesion depth. Wagner Grade ≥2 ulcers are considered intermediate to advanced, involving deeper tissues and increasing risks of infection, osteomyelitis, and amputation. These advanced ulcers precede 85% of diabetes-related nontraumatic lower-limb amputations, with 5-year mortality rates of 40-55%, comparable to or exceeding many cancers. Key risk factors for poor outcomes include advanced age, male, peripheral arterial disease, renal impairment, and ischemic ulcers (6–8).

DFU pathogenesis involves multifactorial mechanisms, primarily peripheral neuropathy leading to unnoticed trauma from repetitive stress, impaired peripheral circulation delaying healing, and foot deformities creating high-pressure areas. Additional contributors to Wagner Grade ≥2 ulcers include long diabetes duration, smoking, obesity, hypertension, low ankle-brachial index, elevated HbA1c, and inflammatory markers such as neutrophil-to-lymphocyte ratio (9, 10). Emerging evidence highlights coagulation and immune dysregulation in diabetic complications (11, 12).

Despite advances in DFU management, including offloading, debridement, and antimicrobial therapy, challenges persist in the early identification and stratification of patients with deeper ulcers. Few existing risk models have incorporated thromboelastography parameters, which enable dynamic assessment of clot formation and fibrinolysis and may offer advantages over traditional static predictors such as glycemic control or inflammatory ratios (12). This study focused on hospitalized patients, a population with a higher prevalence of severe DFUs and an urgent need for rapid risk stratification upon admission. We aimed to develop and validate a diagnostic nomogram for Wagner Grade ≥2 DFUs in hospitalized patients with T2DM, integrating thromboelastography parameters and hematological indicators to support early detection, risk stratification, and clinical decision-making.

## Materials and methods

### Participants

This study followed the Transparent Reporting of a multivariable prediction model for Individual Prognosis Or Diagnosis (TRIPOD) guidelines.

This retrospective cohort study included hospitalized adults diagnosed with T2DM according to the 2025 American Diabetes Association criteria (13). The study was conducted at a tertiary referral center with a specialized multidisciplinary diabetic foot care program. Patients were identified through electronic medical records from the endocrinology department of the Second Affiliated Hospital of Fujian Medical University between January 1, 2023, and December 31, 2025. To ensure sufficient events for robust model development, we deliberately employed a case-enriched sampling approach by including all patients with Wagner Grade ≥2 DFUs during the study period (n=248) and randomly selecting an approximately equal number of controls without DFUs (n=262). DFUs were classified using the Wagner grading system, with Grade ≥2 defined as ulcers extending to tendon, capsule, or bone, including deeper infections or gangrene. Demographic, clinical, thromboelastography (TEG), and other hematological data were collected at admission.

Inclusion criteria were as follows: (1) fulfillment of T2DM diagnostic standards (13), (2) DFUs conforming to Wagner Grade ≥2 (14), (3) minimum age of 18 years, and (4) hospitalization for diabetes-related management or complications with fully documented and verifiable medical histories.

Exclusion criteria were as follows: (1) type 1 diabetes mellitus or other forms of diabetes, (2) women during pregnancy or lactation, (3) active malignancy, severe hepatic or renal failure (eGFR < 30 mL/min/1.73 m²), or autoimmune disorders that could confound coagulation profiles, (4) recent major surgery, trauma, or anticoagulant therapy within 4 weeks prior to admission, and (5) incomplete or unreliable medical records, including missing TEG or blood test results.

For external validation, an independent cohort of 154 hospitalized patients with T2DM was identified through electronic medical records from Quanzhou Southeast Hospital during the same period, including 86 with Wagner Grade ≥2 DFUs and 68 controls. The same inclusion and exclusion criteria were applied to ensure comparability. Data collection adhered to the Declaration of Helsinki principles.

### Data collection

Patient demographic and clinical data were retrospectively retrieved from the hospital’s electronic health records system. Collected variables included baseline characteristics such as age, gender, body mass index (BMI), systolic blood pressure (SBP), and diastolic blood pressure (DBP). Comorbid conditions encompassed hypertension (HBP), coronary artery disease (CAD), stroke, diabetic kidney disease (DKD), and diabetic foot ulcer (DFU). Admission laboratory parameters comprised white blood cell count (WBC), monocyte count (Mono), lymphocyte count (Lymph), platelet count (PLT), fibrinogen (FIB), Angle α, K time, fasting blood glucose (FBG), hemoglobin A1c (HbA1c), alanine aminotransferase (ALT), aspartate aminotransferase (AST), total cholesterol (TC), triglycerides (TG), high-density lipoprotein cholesterol (HDL-C), low-density lipoprotein cholesterol (LDL-C), creatinine (Cr), and uric acid (UA). Data entry and validation were performed independently by two trained investigators to maintain accuracy and consistency.

### Modeling process

All clinical and laboratory data were scrutinized for completeness. Missing data were present in <5% of variables. Multiple imputation by chained equations (MICE) with 5 imputations and 10 iterations was performed; convergence was confirmed by trace plots. Patients were randomly divided into a training set (n=357) and an internal validation set (n=153) in a 7:3 ratio.

Potential predictors were screened using univariate logistic regression analysis. Variables with p < 0.1 in the univariate analysis, along with clinically recognized risk factors, were incorporated into the multivariable logistic regression model. A backward stepwise selection process based on the likelihood ratio test was used to identify independent risk factors for Wagner Grade ≥2 DFUs in patients with T2DM. Multicollinearity among the predictors was assessed using the Variance Inflation Factor (VIF) and tolerance. All VIF values were well below the conventional threshold of 10 and tolerance values were above 0.1, confirming the absence of significant collinearity. With 248 events and 4 final predictors, the events-per-variable (EPV) ratio was 62, well above the recommended threshold of 10. Subsequently, a diagnostic nomogram was constructed based on these independent predictors to provide a personalized risk assessment tool. Model discrimination was evaluated using ROC curves with AUC values and 95% CIs. Calibration curves were employed to assess the agreement between predicted probabilities and observed outcomes. The clinical applicability of the nomogram was further examined through DCA and clinical impact curves, which quantified net benefits across a range of probability thresholds. External validation was conducted in an independent cohort to confirm the model’s generalizability.

### Statistical analysis

All statistical analyses were conducted using SPSS version 26.0 (IBM Corp., Armonk, NY, USA) and R version 4.2.2 (R Foundation for Statistical Computing, Vienna, Austria). Continuous variables conforming to a normal distribution were presented as mean ± standard deviation (mean ± SD), whereas non-normally distributed continuous variables were expressed as median (interquartile range). Categorical variables were reported as frequencies and percentages (n [%]). Between-group comparisons were performed using one-way ANOVA or Kruskal-Wallis test for continuous variables and chi-squared test for categorical variables, with *post-hoc* pairwise testing and Bonferroni correction. The dataset was randomly divided into training and internal validation sets in a 7:3 ratio using the “createDataPartition” function from the “caret” package in R, ensuring balanced distribution of the outcome variable. ROC curves were generated and analyzed using the “pROC” package. Calibration curves, DCA, and clinical impact curves were created with the “ggplot2,” “rmda,” and “regplot” packages. All tests were two-sided, with statistical significance defined as P < 0.05.

## Results

### Clinical characteristics

This study included 510 hospitalized patients with T2DM at the Second Affiliated Hospital of Fujian Medical University, among whom 248 had Wagner Grade ≥2 DFUs. Patients were randomly divided into a training set (n=357) and an internal validation set (n=153). An external validation cohort comprised 154 patients with T2DM from Quanzhou Southeast Hospital, including 86 with Wagner Grade ≥2 DFUs. **Table 1** summarizes the baseline demographic characteristics, vital signs, comorbidities, and laboratory findings across the training, internal validation, and external validation sets.

**Table 1:** General clinical characteristics.

Item	Groups			*P* value
	Training set (n=357)	Internal validation set (n=153)	External validation set (n=154)	
Age (years)	58.88 ± 14.08^c^	60.66 ± 12.39	62.89 ± 11.75^a^	0.006
Gender (%)				0.864
Male	221 (61.90%)	97 (63.40%)	99 (64.29%)	
Female	136 (38.10%)	56 (36.60%)	55 (35.71%)	
BMI (kg/m2)	23.64 ± 4.21^c^	23.55 ± 3.43^c^	24.85 ± 4.00^a,b^	0.023
Wagner Grade 2 or higher DFU (%)				0.631
Yes	172 (48.18%)	76 (49.67%)	86 (55.84%)	
No	185 (51.82%)	77 (50.33%)	68 (44.16%)	
HBP (%)				<0.001
Yes	183 (51.26%) ^c^	88 (57.52%)	98 (63.64%) ^a^	
No	174 (48.74%)	65 (42.48%)	56 (36.36%)	
CAD (%)				<0.001
Yes	75 (21.01%) ^c^	36 (23.53%)	19 (12.34%) ^a^	
No	282 (78.99%)	117 (76.47%)	135 (87.66%)	
Stroke (%)				0.597
Yes	36 (10.08%)	12 (7.84%)	12 (7.79%)	
No	321 (89.92%)	141 (92.16%)	142 (92.21%)	
DKD (%)				<0.001
Yes	121(33.89%) ^c^	47 (25.99%) ^c^	27 (42.86%) ^a, b^	
No	236 (66.11%)	106 (74.01%)	127 (57.14%)	
SBP (mmHg)	132.72 ± 20.46	130.46 ± 19.03 ^c^	136.44 ± 24.50 ^b^	0.043
DBP (mmHg)	78.99 ± 11.17	78.40 ± 11.24	80.38 ± 12.28	0.337
WBC (10^9/L)	9.31 ± 5.25	8.94 ± 4.51	8.31 ± 3.69	0.094
Mono (10^9/L)	6.50 ± 2.31 ^c^	6.41 ± 2.08 ^c^	5.45 ± 6.17 ^a,b^	0.008
Lymph (10^9/L)	23.13 ± 11.75	23.73 ± 11.82	21.96 ± 10.46	0.383
PLT (10^9/l)	267.62 ± 100.17	271.44 ± 107.87	282.31 ± 109.21	0.343
FIB (g/l)	4.58 ± 2.05	4.48 ± 1.73	4.30 ± 1.60	0.303
Angle α (°)	67.42 ± 9.76	68.11 ± 7.83	68.74 ± 6.78	0.273
K time (min)	1.51 ± 0.64	1.45 ± 0.51	1.95 ± 5.81	0.223
FBG (mmol/l)	8.08 ± 3.80 ^c^	8.13 ± 3.27 ^c^	10.38 ± 7.09 ^a,b^	<0.001
HBA1C (%)	8.50 ± 2.32 ^c^	8.63 ± 2.42 ^c^	7.84 ± 2.01 ^a,b^	0.003
ALT (U/L)	21.69 ± 41.10	29.33 ± 53.78	27.07 ± 40.49	0.153
AST (U/L)	20.78 ± 31.87	31.32 ± 111.43	22.51 ± 15.33	0.173
TC (mmol/L)	4.34 ± 1.54	4.42 ± 1.52	4.39 ± 1.53	0.617
TG (mmol/L)	1.79 ± 1.58	1.83 ± 1.90	1.98 ± 1.56	0.478
HDL-C (mmol/L)	1.04 ± 0.43^c^	1.06 ± 0.41 ^c^	1.25 ± 0.19 ^a,b^	<0.001
LDL-C (mmol/L)	2.58 ± 1.19	2.63 ± 1.25	2.79 ± 1.25	0.213
Cr (umol/L)	156.14 ± 174.04	159.66 ± 183.78	121.81 ± 156.56	0.083
UA (umol/L)	324.01 ± 118.06 ^c^	321.75 ± 117.14 ^c^	295.05 ± 117.95 ^a,b^	0.033

n, number of patients; a, significantly different from training set (p < 0.05, Bonferroni-adjusted for multiple comparisons); b, significantly different from internal validation set; c, significantly different from external validation set; Between-group comparisons were performed using one-way ANOVA or Kruskal-Wallis test for continuous variables and chi-squared test for categorical variables, with *post-hoc* pairwise testing and Bonferroni correction; BMI, body mass index; DFU, diabetic foot ulcer; HBP, Hypertension; CAD, Coronary Artery Disease; DKD, Diabetic Kidney Disease; SBP, Systolic Blood Pressure; DBP, Diastolic Blood Pressure; WBC, white blood cells; Mono, Monocyte; Lymph, Lymphocyte; PLT, platelet; Fib, fibrinogen; K time, Kinetics time; FBG, fasting blood glucose; HbA1c, Hemoglobin A1c; ALT, Alanine Aminotransferase; AST, Aspartate Aminotransferase; TC, Total cholesterol; TG, Triglycerides; HDL-C, High-Density Lipoprotein cholesterol; LDL-C, Low-Density Lipoprotein cholesterol; Cr, Serum creatinine; UA, uric acid.

### Univariate and multivariate logistic regression analysis

Univariate logistic regression was conducted on the training set to identify potential risk factors associated with Wagner Grade ≥2 DFUs in patients with T2DM. Significant associations were observed for several variables, including age, BMI, HBP, CAD, stroke, DKD, SBP, DBP, WBC, monocytes, lymphocytes, PLT, Fib, Angle α, K time, FBG, HbA1c, TC, TG, HDL-C, and LDL-C (**Table 2**).

**Table 2:** Univariate and multivariate logistic regression analysis.

Variable	β value	OR (95% CI)	P value
Univariate analysis			
Gender	-0.118	0.889 (0.580-1.363)	0.589
Age	-0.006	0.936 (0.919-0.954)	<0.001
BMI	0.204	1.226 (1.147-1.311)	<0.001
SBP	-0.014	0.986 (0.975-0.996)	0.008
DBP	0.031	1.031 (1.011-1.052)	0.002
HBP	-1.016	0.362 (0.236-0.556)	<0.001
CAD	-2.172	0.114 (0.056-0.231)	<0.001
Stroke	-1.906	0.149 (0.056-0.392)	<0.001
DKD	-1.110	0.330 (0.207-0.525)	<0.001
FBG	-0.124	0.883 (0.829-0.941)	<0.001
HbA1c	-0.161	0.851 (0.773-0.937)	0.001
WBC	-0.272	0.762 (0.698-0.831)	<0.001
Mono	0.099	1.105 (1.006-1.213)	0.036
Lymph	0.159	1.172 (1.135-1.210)	<0.001
PLT	-0.005	0.995 (0.993-0.998)	<0.001
Fib	-0.822	0.440 (0.362-0.533)	<0.001
Angle α	-0.268	0.765 (0.724-0.809)	<0.001
K time	4.512	91.064 (35.288-235.003)	<0.001
ALT	0.001	1.000 (0.995-1.005)	0.957
AST	-0.002	0.998 (0.991-1.006)	0.630
TC	0.032	1.385 (1.177-1.631)	<0.001
TG	0.182	1.200 (1.028-1.399)	0.020
LDL	0.573	1.773 (1.068-2.944)	0.027
HDL	0.408	1.504 (1.240-1.825)	<0.001
Cr	0.001	1.000 (0.998-1.001)	0.571
UA	0.001	1.000 (0.999-1.002)	0.608
Multivariate analysis			
Lymph	0.136	1.146 (1.074–1.223)	< 0.001
PLT	0.005	1.005 (1.000–1.011)	0.037
Angle α	-0.11	0.896 (0.813–0.986)	0.025
K time	1.975	7.205 (1.295–40.091)	0.024

Variables significant in the univariate analysis were entered into a multivariate logistic regression model. Backward stepwise selection based on the likelihood ratio test was applied to identify independent predictors. The final model retained four independent risk factors for Wagner Grade ≥2 DFUs in patients with T2DM, including lymphocytes, PLT, Angle α, and K time (P < 0.05, **Table 2**).

### Development of the diagnostic nomogram

**Figure 1:**
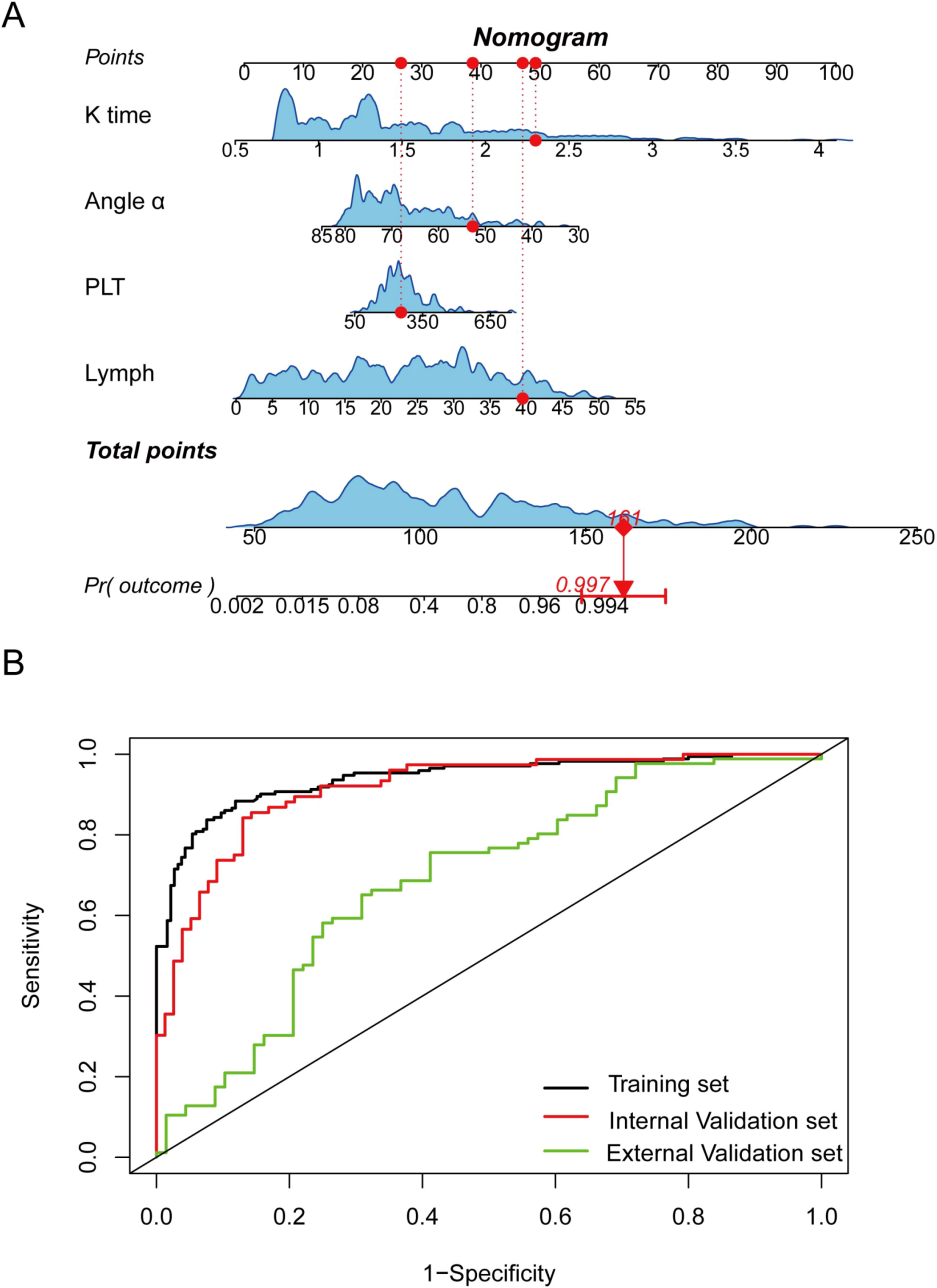
Nomogram and ROC curves for the diagnostic model in patients with Wagner grade ≥2 DFUs. **(A)** Nomogram incorporating K time, Angle α, platelet count (PLT), and lymphocyte count (Lymph) to estimate the probability of adverse outcomes [Pr(outcome)]. Individual variable points are summed to derive total points, which correspond to the predicted risk probability. **(B)** ROC curves demonstrating the model’s discriminatory performance in the training set (black), internal validation set (red), and external validation set (green).

Utilizing the independent predictors derived from multivariate logistic regression, a diagnostic nomogram was constructed to estimate the probability of the presence of Wagner Grade ≥2 DFUs in patients with T2DM. The model integrates K time, Angle α, platelet count, and lymphocyte count, each mapped to a point scale of 0 to 100 (**Figure 1**). To apply the nomogram, individual variable values are aligned with their corresponding points on the upper axes, which are then aggregated to yield a total score (ranging from 50 to 250). This total is projected downward to the probability axis, providing the estimated probability of the presence of Wagner Grade ≥2 DFUs, from as low as 0.2% to 99.4%. This visual tool enables clinicians to perform quick, individualized assessments by drawing straight lines from each variable value to the points axis and aggregating the results. The full multivariable logistic regression equation was: logistic(p) = 2.900 × K time + 0.126 × Lymph + 0.004 × PLT − 0.081 × Angle α − 2.592 (where p is the probability of the presence of Wagner Grade ≥2 DFUs).

### Model performance and validation

The discriminative ability of the diagnostic nomogram was evaluated using ROC curves. In the training set, the AUC was 0.940 (95% CI: 0.916–0.965), indicating excellent discrimination. The internal validation cohort yielded an AUC of 0.914 (95% CI: 0.870–0.956), confirming robust performance. External validation in the independent cohort showed an AUC of 0.690 (95% CI: 0.604–0.775), demonstrating modest discrimination (**Figure 1**).

**Figure 2:**
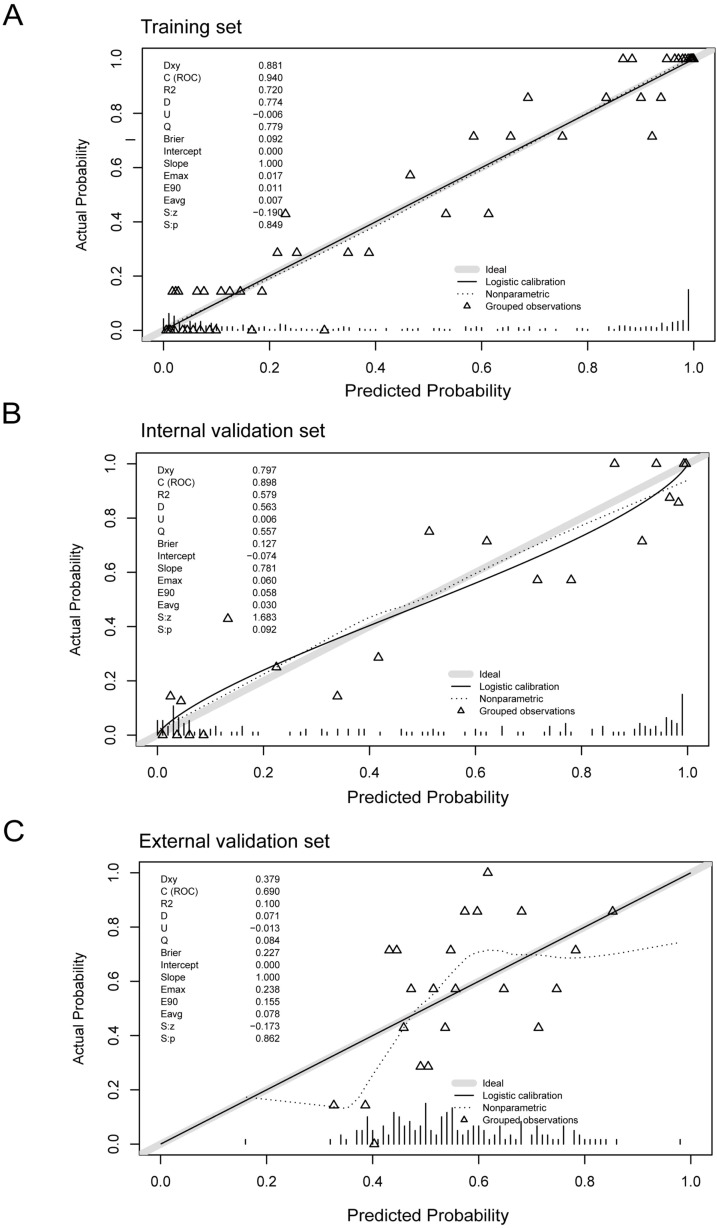
Calibration curves for the training set, internal validation set, and external validation set, with reported metrics (slope, intercept, Brier score, and Emax). **(A)** Training set. **(B)** Internal validation set. **(C)** External validation set.

Calibration assessments were conducted via plots comparing predicted versus observed probabilities (**Figure 2**). For the training and internal validation cohorts, the apparent and bias-corrected curves demonstrated excellent concordance with the ideal line, indicating precise probability estimation without substantial bias (Hosmer-Lemeshow test, P > 0.05). External calibration was assessed visually as suboptimal. Although the calibration slope was ideal (1.000) and the intercept was 0.000 in the external validation cohort, the Brier score was relatively high (0.227) and the maximum calibration error (Emax = 0.238), indicating moderate deviation from perfect calibration. This may reflect population heterogeneity or differences in data distribution between the development and external sets.

**Figure 3:**
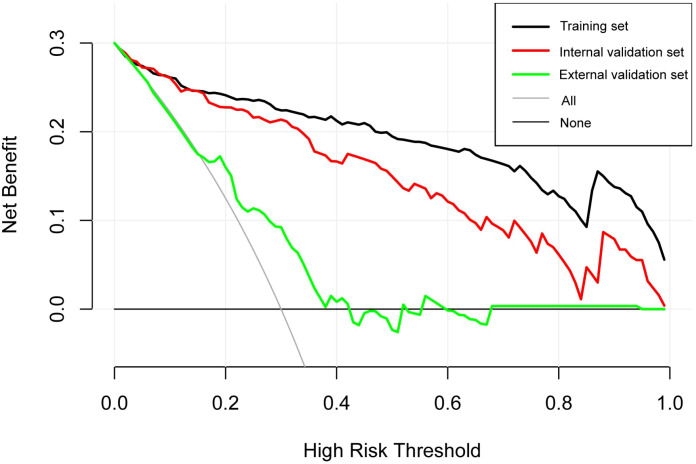
Decision curve analysis (DCA) evaluating the net clinical benefit of the diagnostic nomogram across various threshold probabilities for patients with Wagner Grade ≥2 DFUs. Curves represent the training set (black), internal validation set (red), external validation set (green), all-intervene strategy (gray), and no-intervene strategy (horizontal line at zero).

Clinical utility was examined through decision curve analysis (DCA; **Figure 3**). The nomogram provided net benefits superior to “treat all” or “treat none” approaches across a wide range of threshold probabilities (approximately 0.1–0.9) in all cohorts, including the external validation set, where benefits were still evident despite modest overall performance. This supports the model’s potential to inform clinical decisions by balancing true positives against false positives.

**Figure 4:**
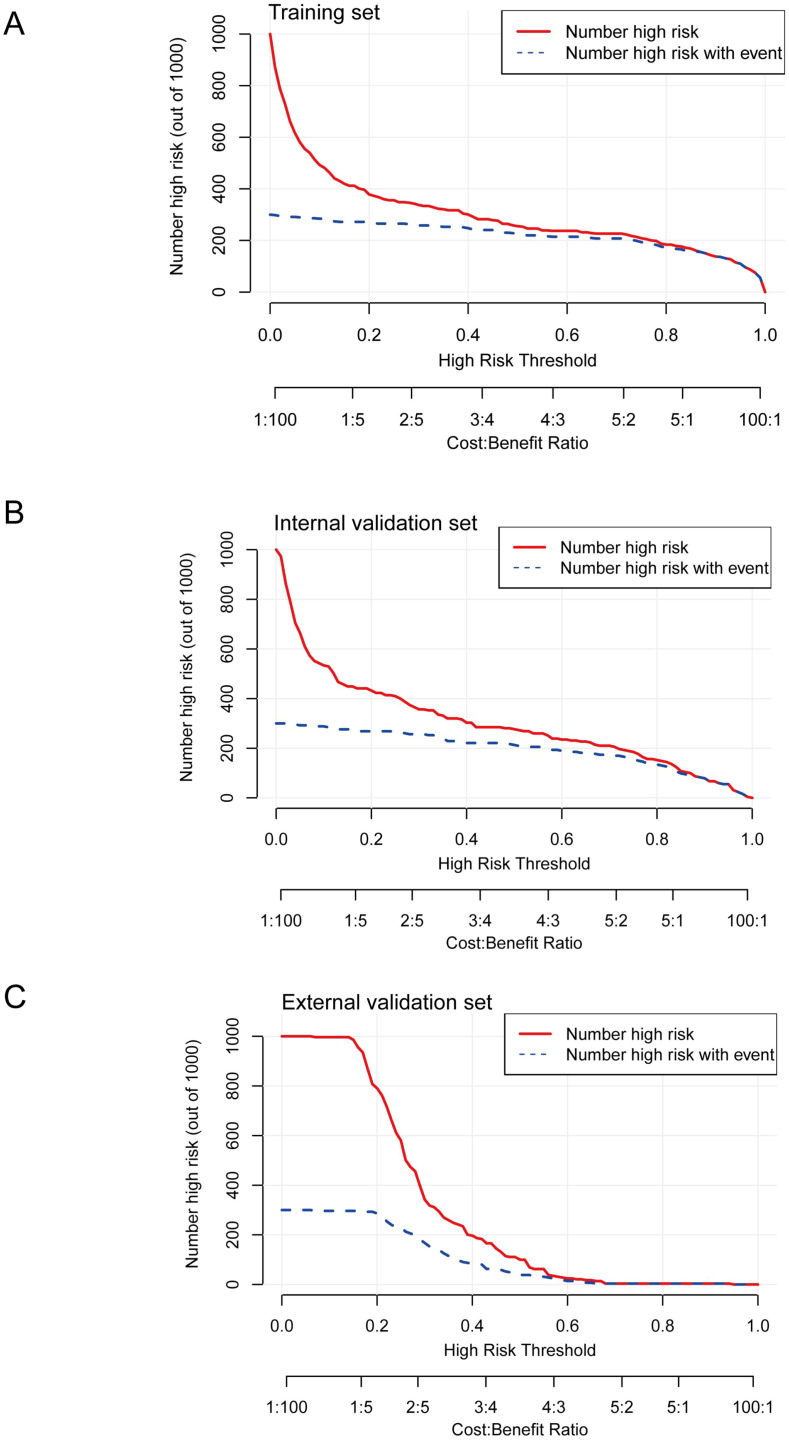
Clinical impact curves depicting the estimated number of patients classified as high-risk and those experiencing actual adverse events per 1,000 individuals at different high-risk thresholds and cost:benefit ratios for the model in Wagner Grade ≥2 DFUs. **(A)** Training set showing number classified as high risk (red solid) and number high risk with event (blue dashed). **(B)** Internal validation set displaying number classified as high risk (red solid) and number high risk with event (blue dashed). **(C)** External validation set illustrating number classified as high risk (red solid) and number high risk with event (blue dashed).

Further, clinical impact curves depicted the projected number of high-risk classifications per 1,000 patients at varying thresholds and cost:benefit ratios (**Figure 4**). In each cohort, as risk thresholds increased, the number of patients flagged as high-risk decreased, with a corresponding drop in true events detected. The training and internal validation sets showed efficient stratification, identifying a high proportion of true positives at lower thresholds (cost:benefit ratios of 1:100 to 1:5). The external validation set exhibited similar trends but with modestly reduced sensitivity for events, consistent with the observed calibration limitations, yet still offering practical value for risk prioritization in real-world applications.

## Discussion

In this study, we developed and validated a diagnostic nomogram incorporating TEG parameters (Angle α and K time) alongside hematological indicators (platelet count and lymphocyte count) to identify the presence of Wagner Grade ≥2 DFUs in hospitalized patients with T2DM. The nomogram demonstrated good discrimination in the training and internal validation cohorts, but showed a reduction in area under the curve (AUC) in the external validation cohort. Several factors may have contributed to this decrease, including potential overfitting associated with backward stepwise selection, between-center differences in patient demographics, disease severity, clinical management, and laboratory conditions, as well as site-specific effects. In addition, inconsistencies in regression directions were observed: age showed a protective effect in univariate analysis but was not retained after multivariable adjustment, whereas platelet count remained statistically significant. These observations underscore the complexity of model development in real-world clinical settings and the challenges of achieving generalizability.

Significant differences in baseline characteristics were observed between the development and external validation cohorts (**Table 1**), including higher fasting blood glucose, lower HbA1c, higher HDL-C levels, and greater prevalence of diabetic kidney disease (all p < 0.05). These differences in case mix likely contributed to the observed reduction in model performance during external validation. Although decision curve analysis indicated net benefit across a wide threshold range (0.1–0.9) in the development cohorts, the benefit was more modest in the external validation cohort, consistent with its lower discrimination. This suggests the nomogram is most useful for risk prioritization rather than definitive diagnosis in heterogeneous populations. Future prospective multicenter studies with larger sample sizes are needed to further validate and refine the model.

The inclusion of TEG parameters, such as Angle α (reflecting fibrinogen and platelet function in clot formation) and K time (indicating time to reach a fixed clot strength), underscores the role of hypercoagulability and impaired fibrinolysis in the pathogenesis of Wagner Grade ≥2 DFUs. These markers align with evidence of altered hemostasis in diabetes, where prolonged K time and reduced Angle α may signal delayed clot kinetics, exacerbating tissue ischemia and ulcer progression (12, 15). Similarly, elevated platelet count and lymphocyte count emerged as independent predictors, consistent with inflammatory pathways in diabetic complications (16). Platelet activation contributes to microvascular thrombosis, while lymphocyte dysregulation reflects chronic immune imbalance, both of which may impair wound healing (17). The platelet-to-lymphocyte ratio (PLR), a composite of these factors, has previously been associated with DFU severity, amputation risk, and mortality (18).

Although TEG parameters were central to our model, TEG is not routinely available in many endocrinology departments and incurs additional costs. This may limit its widespread clinical implementation, particularly in resource-limited or primary care settings. Future studies should explore simplified models using only routine hematological parameters (such as PLT and lymphocyte count) or validate point-of-care TEG alternatives to improve accessibility and practicality.

In comparison to prior models for DFU risk stratification, our nomogram incorporates thromboelastography parameters, whereas many existing approaches rely on clinical indicators such as peripheral vascular disease, glycemic control metrics, or ulcer classification systems (19–24). Our model provides an alternative perspective emphasizing coagulation profiles that may complement existing tools in settings where TEG is available.

This study had several limitations. First, the high prevalence of Wagner Grade ≥2 DFUs (~48.6%) resulted from intentional case-enriched sampling at a tertiary referral center, which may introduce spectrum bias. Second, the substantial drop in external AUC (0.940 to 0.690) and suboptimal calibration indicate possible overfitting, case-mix differences. Third, reliance on TEG parameters may restrict routine clinical use due to limited availability and additional costs. Finally, this model is a cross-sectional diagnostic tool for prevalent Wagner Grade ≥2 DFUs at admission rather than a longitudinal prognostic model for future ulcer development. Future prospective multicenter studies with larger samples and model recalibration are warranted to validate and improve its performance.

## Conclusion

In conclusion, we developed a diagnostic nomogram integrating thromboelastography parameters with routine hematological indicators to identify the presence of Wagner Grade≥2 DFUs in hospitalized patients with T2DM. The model showed excellent discrimination and calibration in the training and internal validation cohorts, with modest performance in the external validation cohort. By incorporating coagulation and inflammatory markers, the nomogram offers a practical tool for risk stratification that may facilitate early clinical decision-making and support efforts to reduce complication rates.

## Data Availability

The original contributions presented in the study are included in the article/supplementary material. Further inquiries can be directed to the corresponding author.
